# Behavioral, Histopathological, and Biochemical Implications of Aloe Emodin in Copper-Aβ-Induced Alzheimer’s Disease-like Model Rats

**DOI:** 10.3390/cimb48010086

**Published:** 2026-01-15

**Authors:** Xitong Zhao, Jianing Yin, Baojian Du, Wenqian Fan, Yang Chen, Yazhu Yang, Fang Fang, Jun Guan

**Affiliations:** School of Chinese Materia Medica, Beijing University of Chinese Medicine, Beijing 102488, China; 20210935072@bucm.edu.cn (X.Z.); 20230935095@bucm.edu.cn (J.Y.); 20220935082@bucm.edu.cn (B.D.); 20220935081@bucm.edu.cn (W.F.); 20210935073@bucm.edu.cn (Y.C.); 20200221162@bucm.edu.cn (Y.Y.); fangf1166@126.com (F.F.)

**Keywords:** Alzheimer’s disease, copper, aloe emodin, amyloid β, cognitive impairment, rat models

## Abstract

Simultaneously inhibiting beta-amyloid protein (Aβ) aggregation and reducing metal ion overload in the brain is a promising strategy for treating Alzheimer’s disease (AD). Aloe emodin (AE) is one of the major components of the traditional Chinese medicine rhubarb. Based on its reported pharmacological effects and its structural affinity for metal ions, this study aims to explore the potential of AE in improving AD pathology. Through the injection of Aβ or copper-Aβ complex in the bilateral hippocampus of rats, we constructed two kinds of nontransgenic animal models. Behavioral tests were used to evaluate cognitive impairment, and the effects of AE on neuronal damage and Aβ deposition were measured via Nissl staining and immunohistochemistry. Furthermore, we detected copper content in the serum and brain tissues as well as some biochemical indexes of Aβ cascade pathology in the brain tissues of model rats to explore the mechanism of action. AE treatment decreased copper accumulation and regulated Aβ metabolism in the brain of model rats, thereby improving Aβ deposition, memory impairment, hippocampal nerve cell damage, and related biochemical indicators. AE ameliorated the AD pathology of the model rats by targeting copper-induced Aβ toxicity, revealing a mechanism of action by which AE may exhibit good clinical efficacy in treating AD.

## 1. Introduction

Alzheimer’s disease (AD) is the most common form of dementia and affects millions of people worldwide [1]. The brain changes that stem from AD are characterized by the accumulation of beta-amyloid proteins (Aβ), phosphorylated tau that causes synapse damage and progressive neurodegeneration, which eventually lead to memory loss, cognition decline, language deficiency, and behavioral changes [2,3]. The amyloid cascade hypothesis of AD was first proposed by Hardy and Allsop in 1991 and posits that the accumulation of Aβ is the primary factor that drives AD neurodegenerative downstream events, including oxidative stress (OS), inflammation, and neurotransmitter dyshomeostasis [4,5]. Therefore, inhibiting the imbalance between production and clearance of Aβ has become the most extensively validated and compelling therapeutic target [6].

In addition to the above pathological features, millimolar concentrations of redox active metal ions, including copper, iron, zinc, and aluminum, have been reported in the senile plaques in the brains of AD patients [7]. Increased copper in the brain is strongly related to the modulation of cognition [8], as such unregulated redox active metal ions generates high levels of OS, thereby leading to neuronal loss [9]. Furthermore, copper significantly affects the amyloid cascade, where it increases Aβ loading by regulating its clearance and/or production [10], and ultimately induces Aβ toxicity [11]. Copper not only promotes Aβ pathology but also binds to Aβ directly to form stable complexes [12]. However, it is widely believed that the aggregated Aβ, in concert with complexed redox metal ions, produces free radicals and catalyzes the formation of reactive oxygen species [13]. Due to its peroxidase activity and redox properties [14], these copper-Aβ complexes result in a higher neurotoxicity than metal-free Aβ [15]. Therefore, simultaneous inhibition of Aβ aggregation and reduction in metal ion overload in the brain are a promising strategy for developing therapeutic agents for AD [16].

This kind of best drug candidates classify into two multifunctional mechanisms: antioxidant agents, molecules whose standard reduction potentials are high enough to diminish the oxidative stress in the brain caused by the copper-induced hydrogen peroxide generation cycle; and cooperation agents, ligands meant to regulate copper levels at brain’s level by distributing these ions into the neurons [17].

Traditional Chinese medicine (TCM) has demonstrated its effectiveness in treating neurological disease over its long history of empirical clinical application in China [18]. According to the TCM theory of “brain collateral damage due to toxins”, rhubarb (Rhei Radix et Rhizoma) has produced excellent therapeutic effects for AD through its actions of “activating blood circulation” and “dissolving blood stasis” [19]. One of the major anthraquinone components of rhubarb, aloe emodin (1,8-dihydroxy-3-hydroxymethyl-anthraquinone, AE), is a potential candidate for the treatment of neurodegenerative disease due to its pharmacological effects, including antioxidant, anti-inflammatory, and neuroprotective capabilities [20]. Studies focused on the pharmacological mechanism of AE have shown that AE depends on scavenging hydroxyl radicals to exert antioxidant potency and that the polar hydrophilic hydroxymethyl group substitution possibly contributes to its anti-inflammatory capacity [21].

There is also a chemical structural advantage inherent to AE (Figure S1). Since it contains electronegative 1-/8-hydroxyl and 9-carbonyl oxygen atoms, AE has high metal binding affinities to coordinate copper ions and as a result may be able to regulate the homeostasis of metal ions in AD brains as a specific metal ligand. In fact, a molecular docking simulation study illustrated that anthraquinone derivatives can interact with metal binding sites at the N-terminal on Aβ, thus significantly interfering with Cu^2+^-induced Aβ aggregation [22]. Moreover, AE has been found to exhibit a strong inhibitory effect on Aβ self-aggregation [23], primarily due to intercalation into the β-sheet and destabilization of interstrand hydrogen bonds [24].

AE has been found to pass through the blood–brain barrier (BBB) in the brain of rats [25]. Considering that molecules that possess strong metal coordination and Aβ interaction capacity have long been explored as AD therapeutics [26], we hypothesize that AE can inhibit Aβ toxicity by interacting with copper ions and interfering with the stability of copper-Aβ species (the possible mechanism is shown in Figure S2), thereby alleviating the neurotoxicity and improving the cognitive impairment. Although mechanistic studies have focused on the pharmacological effects of AE, there is scant mechanistic research that has addressed the effects of AE on the Aβ pathological cascade from the perspective of metal coordination. Our study thus aimed to elucidate the mechanism of AE on behavioristics, histopathology, and histochemistry of AD-like rats from the perspective of metal coordination.

## 2. Materials and Methods

### 2.1. Materials

Amyloid Beta 1-42 Rat (E1375) was purchased from Sigma Aldrich (St. Louis, MO, USA). Aloe emodin (A17GS145379, HPLC purity ≥ 95%) was purchased from Shanghai Yuanye Biotechnology Co., Ltd. (Shanghai, China). Anhydrous copper (II) chloride (C14071629, 99.99% metals basis) was purchased from Shanghai Macklin Biochemical Technology Co., Ltd. (Shanghai, China). Donepezil hydrochloride was purchased from Eisai China Inc. (2110031, Shanghai, China).

### 2.2. Animals

Specific pathogen-free (SPF) healthy male SD rats, weighing 200−220 g (6 weeks of age), were obtained from the SPF Biotechnology Co., Ltd. (Beijing, China) and raised in the SPF animal laboratory of Experimental Animal Center of Beijing University of Chinese Medicine [(22 ± 2) °C, 55%−65% humidity, 12 h/12 h light–dark illumination cycle]. All animal experiments were conducted in accordance with ethical animal research standards of the Chinese Council on Animal Care and approved by the Ethics Committee of Experimental Animals of Beijing University of Chinese Medicine (permit number: BUCM-2023091303-3156; approval date: 18 September 2023).

### 2.3. Preparation of the Solutions

The Aβ solution: To prevent the presence of any preformed aggregates, Aβ monomer was dissolved in precooled 1,1,1,3,3,3-hexafluoroisopropanol (HFIP) with a concentration of 1 g/L [27]. HFIP was removed by termovap sample concentrator (N-EVAP 116, Organomation, Worcester, MA, USA). The formed visible colorless peptide film was stored at −20 °C until used. When used, solid Aβ monomer peptide film was initially dissolved in dimethyl sulfoxide (DMSO) and supplemented with phosphate-buffered saline (PBS, 1X) with a final concentration of 5 g/L. Keep the solution in a constant temperature shaking incubator for 7 days at 37 °C.

The Cu-Aβ solution: The copper-Aβ complex was prepared in a 1/1 metal/peptide ratio. First, anhydrous CuCl2 was dissolved in DMSO and supplemented with PBS with a final concentration of 0.3 g/L (CuCl_2_ solution). The solid Aβ monomer peptide film was dissolved in DMSO and supplemented with PBS with a final concentration of 10 g/L, then 100 μL CuCl_2_ solution was added. The mixed solution was kept in a constant temperature shaking incubator for 7 days at 37 °C.

The AE solution: AE solid powder was fully dissolved with DMSO, then Tween 80 was added, and finally diluted to an appropriate concentration with 0.9% NaCl to make sure the final content of DMSO and Tween 80 was 10%.

The donepezil solution: Donepezil hydrochloride was fully dissolved in 0.9% NaCl to make a solution of 0.2 g/L.

### 2.4. The Construction of Animal Models

A schematic of the experimental procedure is shown in Figure S3. A schematic of the experimental timeline is shown in Figure S4. A total of 64 rats was randomly divided into eight following groups (eight rats in each group): control group, sham group, rats received a bilateral intrahippocampal injection of Aβ monomer (hippo-Aβ group), rats received a bilateral intrahippocampal injection of copper-Aβ complex (hippo-CuAβ group), hippo-CuAβ rats treated with AE (low-dose AE group, medium-dose AE group, and high-dose AE group) and hippo-CuAβ rats treated with positive drug (donepezil group). Before the surgery, the rats in all groups fasted overnight and were anesthetized with 1% pentobarbital sodium (i.p., 0.5 mL/100 g bw). The head was shaved around the fontanelle region and a 1.5 cm incision was made using surgical scissors for drilling two holes on the skull. Accurate coordinates (3.0 mm after anterior fontanelle, 2.0 mm next to the midline of the left and right brain, and 3.0 mm under the surface of the skull) were determined with the help of brain stereotaxic apparatus (SAS-44611, ASI, Eugene, OR, USA). The rats in the control group received no treatment; the sham group received PBS solution containing 20 vol % of DMSO; the hippo-Aβ group received 2 μL Aβ solution; the five other groups received 2 μL Cu-Aβ solution. In each injection, the solution was delivered at a rate of 1 μL/min which was left in place for 5 min to avoid flow back of the solution after each injection. Use absorbable sutures for wound suturing and maintain aseptic operation throughout the process. After that, apply bupivacaine to the wound for local anesthesia to prevent severe pain. The whole operation time was maintained at about half an hour for each rat. Then, the rats were kept in the single cage at 37 °C until they were completely conscious and able to move independently, which was approximately one hour after anesthesia. After that, the state and body weight of the rats were continuously evaluated. For individuals with poor appetite, glucose water was supplemented in a timely manner.

### 2.5. Animal Administration

Rats were individually weighed on the day of administration, and the exact volume of drug solution for each rat was calculated accordingly. The low-dose, medium-dose, and high-dose AE groups were given three different concentrations of AE solutions (i.p., 3 mg/kg, 6 mg/kg, and 12 mg/kg) per day, which were referred to in previous articles [28]; the donepezil group received donepezil hydrochloride per day (i.g., 0.5 mg/kg); and the other groups were administered mixed solution (0.9% NaCl/DMSO/Tween 80, 8/1/1, *v*/*v*/*v*) for parallel control (i.p., 5 mL/kg). The administrations were performed from 8:30 to 10:30 per day and lasted for 28 days.

### 2.6. Behavioral Tests

#### 2.6.1. Y-Maze Test (YMT)

The YMT was conducted in a Y-shaped apparatus (50 cm long, 10 cm wide, and 30 cm high) with three arms [29]. An entry was defined as when a rat enters the arm of the maze with all four paws, while the sequence of entries in each arm and spontaneous alternations (rats visit the three arms one after the other with a frequency significantly greater than 50%) were recorded. Results were expressed as the percentage (%) of correct alternation (entry into all three arms on consecutive choices)/(total number of arm entries − 2).

#### 2.6.2. Open Field Test (OFT)

The OFT was conducted in a black square open acrylic box of 100 × 100 × 40 cm [30]. Each rat was initially placed at the center of box bottom and allowed to freely explore for 5 min in a quiet environment with dim light. The locomotor activity of rat was recorded and analyzed with EthoVision XT 15 (Noldus, Wageningen, The Netherlands).

#### 2.6.3. Morris Water Maze Test (MWM)

The MWM (including place navigation and spatial probe test) was conducted with the protocol adapted from ref. [31]. The water maze was a black circular pool (diameter 150 cm, height 60 cm) which was filled with temperature-controlled water (24 °C, stained black). A submerged (1 cm) escape platform (diameter 12 cm) was fixed in the center of one of the four equal quadrants (target quadrant). Rats were lowered into the pool in a fixed order at four different starting positions for four days. The time for rats to escape from water onto platform was calculated in seconds (escape latency time, ELT) as the index of acquisition or learning. On the fifth day, the submerged platform was removed from the pool, and the movements of rats were analyzed with the tracking system EthoVision XT 15 (Noldus, Wageningen, The Netherlands) by calculating the amount of time spent in the target quadrant (TSTQ) and platform region (TSPR), as well as the number of times rats crossed the platform region (NTPR).

### 2.7. Sample Preparation

After the completion of behavioral studies, all the animals were fasted overnight and sacrificed. After deep anesthesia with pentobarbital sodium (i.p., 1%, 5 mL/kg), the blood samples were first obtained from all the eight rats in each group through the abdominal aorta. All the blood samples were allowed to clot at room temperature for 1.5 h and then centrifuged (2500 rpm, at 4 °C for 15 min) using a high-speed, refrigerated microcentrifuge (5417R, Eppendorf, Hamburg, Germany). The serum samples were cryopreserved at −80 °C.

Then, three rats in each group were randomly selected to prepare paraffin slices of brain (used for Nissl staining and immunohistochemistry), and the remaining five rats in each group were used to obtain brain tissues. After trans-cardiac perfusion with 0.9% NaCl and paraformaldehyde (PFA, 4%), the whole brain of three rats was removed rapidly and soaked into PFA for fixations. After 24 h of fixation in PFA, the brains were dehydrated in an alcohol gradient (JJ-12J, Junjie, Wuhan, China) and immersed in wax solution. Then, the brain tissue was placed in the embedding machine (JB-P5, Junjie, Wuhan, China) and cooled at −20 °C. After the wax has solidified, the wax block was moved to paraffin microtome and sectioned serially at the thickness of 3 μm by a microtome (RM2016, Leica biosystems, Nussloch, Germany). The remaining five rats were transcardially perfused with 0.9% NaCl solution until remaining blood in the body was flushed out. The whole brain was quickly stripped off on ice, divided and weighed, and then stored at −80 °C until use. The entire sampling operation lasted approximately 5 to 10 min for each rat. During the operation, maintain the environmental temperature at 22 ± 2 °C and the humidity at 55–65%.

### 2.8. Histopathology Assessment

#### 2.8.1. Nissl Staining

Three out of eight rats in each group were randomly selected to retain intact brain tissue for sectioning. We first precisely limited the scope of the systematic analysis to the coronal plane area. And, according to the systematic random sampling method, one section (with a thickness of 3 μm) was randomly taken at 200 μm intervals. A total of three sections were taken from each brain tissue to ensure coverage of different anterior and posterior layers of the hippocampus. Then, as previously described [32], the sections were hydrated by serial dilutions of ethyl alcohol and washed using running water. After staining with toluidine bule for 5 min, the sections were baked dry in a hot air oven (GFL-230, Labotery, Tianjin, China), followed by xylene transparency for 5 min. Then, the sheets were sealed with neutral balsam and observed under upright microscope (Eclipse E100, Nikon, Tokyo, Japan). The imagines in CA1, CA3, and dentate gyrus (DG) hippocampal areas of rats were intercepted and analyzed to detect neuronal morphological changes with the CaseViewer 2.4.0 software (3DHISTECH, Budapest, Hungary) and the number of positive cells was manually calculated using the ImageJ 1.54g software (National Institutes of Health, Bethesda, MD, USA). For the same animal, the positive cell numbers of each subregion on all three sections at different depths were averaged.

#### 2.8.2. Immunohistochemistry (IHC)

Using the procedure adapted from ref. [33], the paraffin slices were dewaxed, hydrated by gradient concentrations of ethanol solutions, immersed in an appropriate amount of citric acid repair solution (pH 6.0), and subjected to heat-induced antigen retrieval for 15 min (8 min at medium heat, 8 min interval and 7 min at medium–low heat). After being cooled naturally, the tissue slices were washed with PBS (pH 7.4) three times for 5 min each and blocked for 30 min with 3% bovine serum albumin (BSA, EZ2921B398, Biofroxx, Einhausen, Germany). Then, the tissues incubated overnight at 4 °C with diluted primary antibody (rabbit anti-Aβ polyclonal antibody, 50 μL, 1:800, 71-5800, Thermo Fisher, Waltham, MA, USA) which was covered evenly, followed by secondary antibody (horseradish peroxidase-labeled goat anti-rabbit IgG antibody, 50−100 μL, 1:500, 5220-0336, SeraCare, Milford, CT, USA) application at ambient temperature for approximately 50 min. After they were sealed with neutral balsam, the sections were photographed with the CaseViewer software (3DHISTECH, Budapest, Hungary). The area ratio of positive areas of Aβ was analyzed in each group of images using ImageJ.

### 2.9. Measurement of Copper Content

The measurement of copper content in brain tissues and blood serum samples was performed using the Inductively Coupled Plasma Mass Spectrophotometer (ICP-MS). The gradient calibration samples were prepared by diluting copper standard solution prepared from National Certified Reference Materials (Copper in Water, 23DA0057, Guobiao Testing & Certification Co., Ltd., Beijing, China). The internal standard solution was prepared with a mixed standard containing six kinds of metal elements (BWT30017-100-N-100, Tan-Mo Technology Co., Ltd., Changzhou, China). The qualified standard curve was determined first by plotting the ratio of instrument response value of copper element in standard solution to germanium element (y) versus the concentrations of copper standard solution (x), which requires the correlation coefficient ≥0.999. We also investigated the detection limits (LOD), accuracy (spike recovery), and precision [relative standard deviation (RSD)].

#### 2.9.1. Serum Copper Content

To prevent anticoagulants used for plasma collection from being contaminated by trace elements and ultimately affecting the assay procedure, we analyzed the copper content from the serum samples [34]. For sample preparation, internal standard solution was added to serum. After it was sonicated, the sample solution was filtered through a filter membrane with a pore size of 0.22 μm. Blank solution was carried out in the same way, without serum. The total content of copper in the serum was determined using ICP-MS unit (iCAP RQ BRE731416, Thermo Fisher, Waltham, MA, USA) and the instrument was calibrated using ICP-MS iCAP Q/RQ Tune Solution (Alfa Aesar 91022278, Thermo Fisher, Mumbai, India). The content of copper in blood serum was calculated based on the standard curve.

#### 2.9.2. Brain Copper Content

Brain samples were further digested by the microwave digestion system (MARS X-press, CEM Corporation, Matthews, NC, USA) for 2 h at 80 °C, and were kept for 2 h at 120 °C; they were put through elevated heating for 4 h at 160 °C. After acid solution was removed, the internal standard solution was added to samples. The blank solution was carried out in the same way, without brain. The total content of copper in the brain was determined using another ICP-MS unit (8900, Agilent, Santa Clara, CA, USA) and the instrument was calibrated using ICP-MS Stock Tuning Solution (5188-6564, Agilent, Santa Clara, CA, USA). The content of copper in brain tissues was calculated based on the standard curve.

### 2.10. Western Blot

The brain tissues were homogenized in an RIPA protein lysis buffer with high-speed low-temperature homogenizer and centrifuged (12,000 rpm, at 4 °C for 10 min) using high-speed, refrigerated microcentrifuge (5417R, Eppendorf, Hamburg, Germany). After they were boiled, the samples were size-fractioned by SDS-PAGE at 140 v. After transferring to the PVDF membranes (pore size 0.45 μm, IPVH00010, Millipore, Bedford, MA, USA), the protein samples were blocked with 5% non-fat dry milk solution. Afterwards, the membranes were immunoblotted with primary antibodies, including purified mouse anti-APP antibody (1:1000, 806005, Biolegend, San Diego, CA, USA), BACE1, RAGE, and LRP-1 rabbit polyclonal antibody (1:500, bs-0164R, bs-0177R and bs-10920R, Bioss, Beijing, China). β-actin mouse monoclonal antibody (1:2000, UM4001, Utibody, Tianjin, China) was used for internal reference. Subsequently, the membranes were incubated with the corresponding secondary antibodies (1:4000, horseradish peroxidase-labeled goat anti-mouse IgG, S0002, Affinity Biosciences, Golden, CO, USA; 1:4000, horseradish peroxidase labeled goat anti-rabbit IgG, S0001, Affinity Biosciences, Golden, CO, USA). The protein signals were visualized by autoradiography (Tanon 5200, Tanon, Shanghai, China). The resultant images were quantified by gray intensity values using the Gel-Pro 32 1.0.2 Analyzer software (Media Cybernetics, Rockville, MD, USA), where the gray intensity value of the internal reference was set as 100%.

### 2.11. Biochemical Assays

Brain tissues were added to the precooled NaCl solution (1/9, *w*/*v*), homogenized in ice-water bath, and centrifuged (3000 rpm, at 4 °C for 10 min). Superoxyde dismutase (SOD, A001-3-2) activity, total antioxidant capacity (T-AOC, A015-2-1), and malondialdehyde (MDA, A003-1-2) level were determined using assay kits provided by the Nanjing Jiancheng Bioengineering Institute (Nanjing, China). The expression of pro-inflammatory cytokines and neurotransmitters were determined by the enzyme-linked immunosorbent assay (ELISA) kit (IL-1α, KT3069-A; IL-1β, KT2923-A; IL-6, KT3066-A; TNF-α, KT3056-A; ACh, KT3392-A; 5-HT, KT3318-A; Glu, KT3476-A; GABA, KT3317-A), which were obtained from Jiangsu Kete Biotechnology Co., Ltd. (Nanjing, China).

### 2.12. Statistical Analysis

Statistical analysis in this study was performed using the SPSS software (Version 20.0, IBM Corp., Armonk, NY, USA). The GraphPad Prism software (Version 10, Dotmatics, Boston, MA, USA) was used for visualization purposes. The measurement data was presented as the mean ± standard deviation. Data with multiple independent tests were processed by one-way analysis of variance (ANOVA), followed by the LSD test for post hoc multiple comparison between-group analyses. The results of place navigation test in MWM were analyzed using two-factor analysis of variance with repeated measures. The Kruskal–Wallis’s test was used to evaluate nonparametric data. Data were considered statistically significant if *p* < 0.05.

## 3. Results

### 3.1. AE Inhibits the Cognitive Impairment of AD-like Model Rats

#### 3.1.1. Y-Maze Test

**Figure 1:**
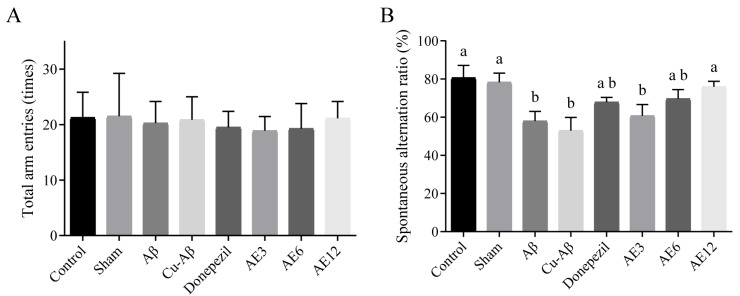
Short-term spatial memory of rats in each group evaluated by the spontaneous alternation ratio in the Y-maze test. (**A**) Total arm entries. (**B**) Spontaneous alternation ratio. Mean values with the same superscript letters are not significantly different, whereas those with the different superscript letters are significantly (*p* < 0.05) different (*n* = 8). According to the concentrations of AE administration, the low-dose AE group is represented by AE3 in the figure, the medium-dose AE group is represented by AE6, and the high-dose AE group is represented by AE12. The following figures are the same.

The total number of arm entries during the test (Figure 1A) showed no significant difference among all groups, which indicates that the general motor activity of rats in this test was not affected by injection and drug administration. In Figure 1B, it was non-significantly different to the spontaneous alternation ratio between the control group and sham group, which demonstrated that molding operation does no harm to the short-term spatial memory of rats. In hippo-Aβ and hippo-CuAβ groups, the spontaneous alternation ratio had considerably decreased as compared to the sham group (25.92%, 32.40%, both *p* < 0.05, Figure 1B), which demonstrated that the cognitive status of model rats was affected more seriously in hippo-CuAβ rats. Although the other groups exhibited a tendency to increase the spontaneous alternation ratio compared with hippo-CuAβ groups (donepezil group, 28.32%; low-dose AE group, 14.76%; medium-dose AE group, 31.53%; high-dose AE group, 43.55%, Figure 1B), only the increase in the high-dose AE was significant (*p* < 0.05). It confirmed that high-dose AE administration was able to reverse the impairment of short-term spatial memory on rats affected by the intracerebral injection of the copper-Aβ complex, which was better than donepezil.

#### 3.1.2. Open Field Test

In the test, the two model groups showed less frequency and less duration observably in the center zone (both *p* < 0.05, Table 1), indicating the anxiety of hippo-Aβ and hippo-CuAβ rats. Compared with the hippo-CuAβ group, the donepezil and medium-dose of AE treatment resulted in relief of rats’ anxiety, and, more concretely, a higher number of entries (both *p* < 0.05) and more duration in the center zone (no statistical difference), whereas there were significant differences between the high-dose group and hippo-CuAβ group in frequency and duration (both *p* < 0.05). Also, there was a significantly better performance for high-dose AE than the donepezil group in frequency index (*p* < 0.05). These data indicated that CuAβ-induced AD-like model animals had anxious-like behaviors that were alleviated by AE treatment, especially by the high dose.

#### 3.1.3. Morris Water Maze Test

**Figure 2:**
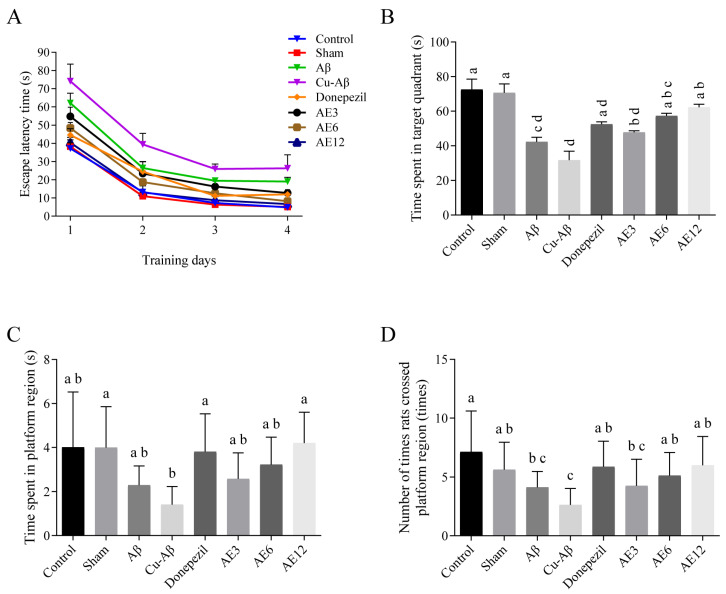
Ability of acquisition learning and memory consolidation of rats in each group evaluated by the escape latency time, the time spent in the target quadrant, the time spent in the platform region, and the number of times rats crossed the platform region. (**A**) Escape latency time. (**B**) Time spent in target quadrant. (**C**) Time spent in platform region. (**D**) Number of times rats crossed platform region. Mean values with the same superscript letters are not significantly different, whereas those with the different superscript letters are significantly (*p* < 0.05) different (*n* = 8).

As shown in Figure 2A, there was a significant decrease in escape latency time (ELT) on second, third, and fourth day sessions compared to the first in all groups, which could be considered as successful acquisition learning due to the four-day training. The time spent in target quadrant (TSTQ) where a hidden platform had previously been placed, the time spent in platform region (TSPR), and the number of times rats crossed platform region (NTPR) by rats searching for the platform were noted as indexes of retrieval in the spatial probe test. There was no significant difference between the control group and sham group in ELT of the place navigation test, as well as the three indexes of the probe test, indicating the absence of the per se effect of operation on acquisition and retrieval (Figure 2). The hippo-CuAβ model group showed a significant difference in ELT, TSTQ, TSPR, and NTPR (all *p* < 0.05) compared with sham group, and the differences were greater than the hippo-Aβ group (Figure 2). It is suggested that the copper-Aβ complex caused severe damage to hippocampal function, which impairs acquisition during hidden platform training trials and subsequent probe trial performance. As shown in Figure 2A, administration of AE in all doses and donepezil significantly prevented the CuAβ-induced increase in the ELT (all *p* < 0.05), and there was an even better performance in high-dose AE than donepezil. Moreover, in the spatial probe test with platform removed, donepezil increased TSPR (*p* < 0.05) and NTPR (*p* < 0.05), compared to the hippo-CuAβ group, and all doses of AE groups improved the three indexes to varying degrees, while the high-dose AE had a more significant difference (all *p* < 0.05, Figure 2B–D). In general, the injection of Aβ or copper-Aβ complex could significantly increase ELT in the place navigation test and decrease probe behaviors. It is represented a severe damage in the process of new memory acquisition and subsequent memory retrieval in the hippo-CuAβ group, while AE greatly improved the memory function of model rats.

**Figure 3:**
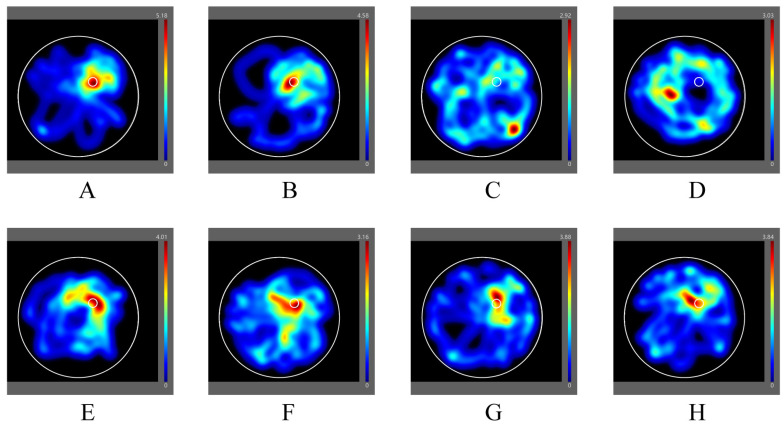
Heatmaps of rats in each group. (**A**) The control group; (**B**) the sham group; (**C**) the hippo-Aβ group; (**D**) the hippo-CuAβ group; (**E**) the donepezil group; (**F**) the low-dose AE group; (**G**) the medium-dose AE group; (**H**) the high-dose AE group (*n* = 8).

Also, swimming distance and swimming tropism were in the target quadrant of donepezil, and AE groups had an increase compared to two model groups, as the heatmaps (Figure 3) showed, which was generated by software to provide a sense of where the animal spent time and the mobility of the animal. These results proved that AE alleviated learning and memory impairments in CuAβ-induced AD rats, especially with a high dose.

### 3.2. AE Ameliorates Neuronal Apoptosis in Hippocampus of AD-like Model Rats

**Figure 4:**
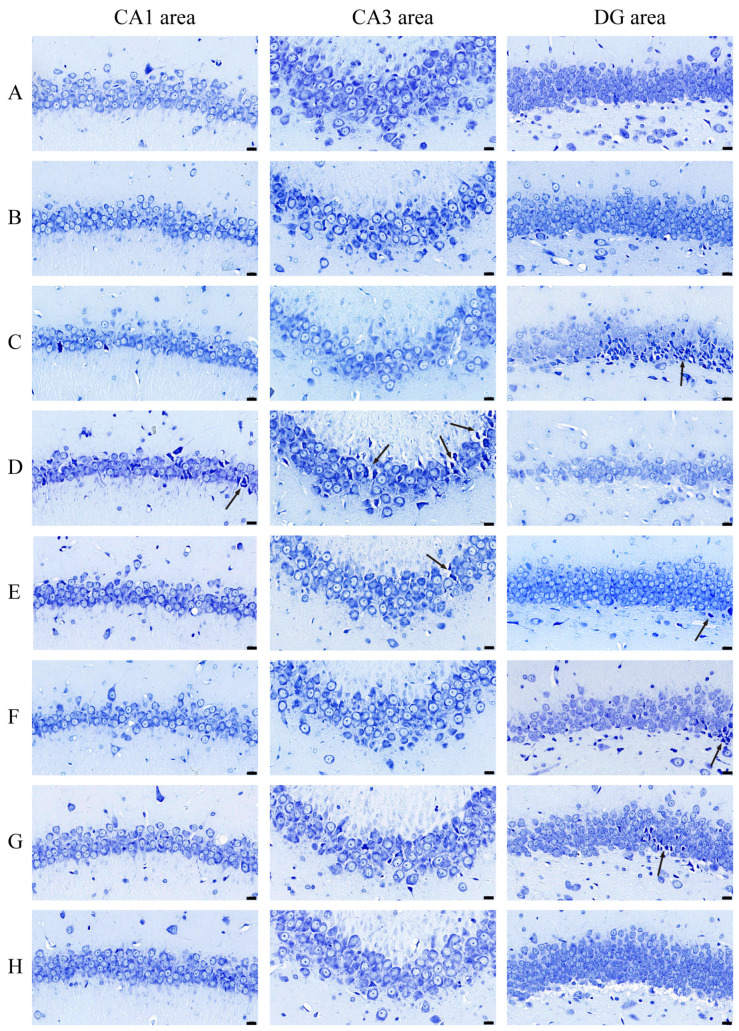
Nissl staining of coronal hippocampus of rats in each group. Scale = 20 μm; magnification × 400. (**A**) The control group. (**B**) The sham group. (**C**) The hippo-Aβ group. (**D**) The hippo-CuAβ group; (**E**) The donepezil group. (**F**) The low-dose AE group; (**G**) The medium-dose AE group; (**H**) The high-dose AE group (*n* = 3).

As shown in Figure 4A,B, there were well-arranged cells, distinct nucleus, abundant Nissl bodies, and intensely stained cytoplasm in the control group and sham group, whereas there was severe neuronal damage in model groups which was manifested by overt neuronal cell loss, vacuolated cytoplasm, and reduced staining intensity (Figure 4C,D). Particularly in the DG area of the hippo-Aβ group and in the CA1 and CA3 area of the hippo-CuAβ group, some neurons appeared distinctly pyknotic (black arrows, darkly stained and closely packed), which illustrated that degenerative changes occurred in the hippocampus of model rats. This finding is consistent with previous research, showing that CA1 and CA3 regions showed higher vulnerability in the transgenic mouse model, characterized by a rapid onset of amyloid pathology [35]. Compared with the hippo-CuAβ group, donepezil and all dose of AE treatments reversed these pathologic changes in a significant manner with a decrease in neurons loss and better stained Nissl bodies. Although there were still some shrunken cells in the DG area of the donepezil group, low and medium dose of the AE group (Figure 4E–G, black arrows), and the degrees of damage were all mitigated. It has been suggested that the DG area is more heavily regulated by various factors than other areas of the hippocampus, due to its capacity for neurogenesis [36]. This may explain why there is less neuronal improvement in the DG area after specific drug treatments. Or, perhaps, AE demonstrates preferential protection of CA1 and CA3 areas. Since a comprehensive understanding of how AE exerts its effects on distinct hippocampal areas remains elusive, this finding warrants further investigation. Nonetheless, the high dose of AE performed better than all the other administration groups, even donepezil. All in all, AE could alleviate the neuronal apoptosis response in the hippocampus of copper-induced AD-like model rats.

### 3.3. AE Reduces the Deposition of Aβ in Hippocampus of AD-like Model Rats

**Figure 5:**
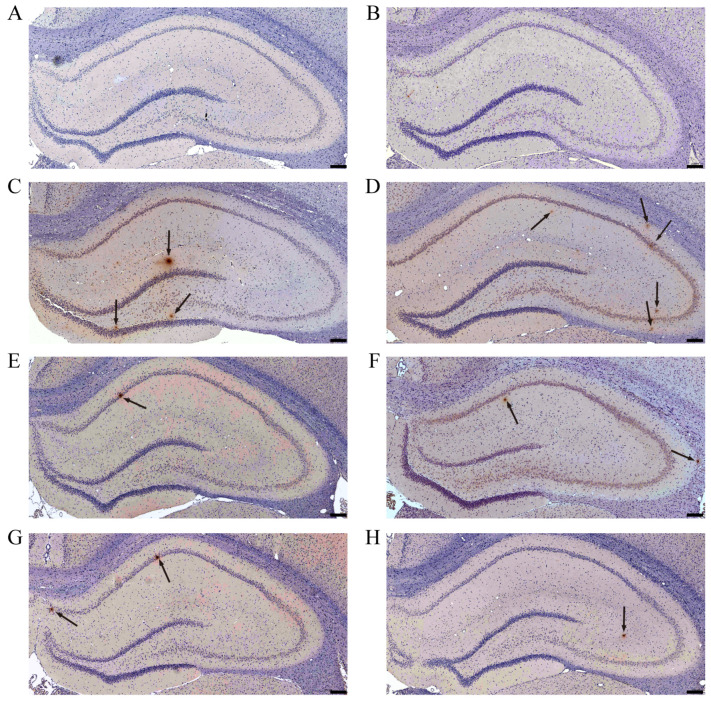
Immunohistochemical plot of Aβ in the rat hippocampus. Scale = 200 μm; magnification × 40. (**A**) The control group. (**B**) The sham group. (**C**) The hippo-Aβ group. (**D**) The hippo-CuAβ group. (**E**) The donepezil group. (**F**) The low-dose AE group. (**G**) The medium-dose AE group. (**H**) The high-dose AE group (*n* = 3). The black arrow indicates the Aβ deposits.

**Figure 6:**
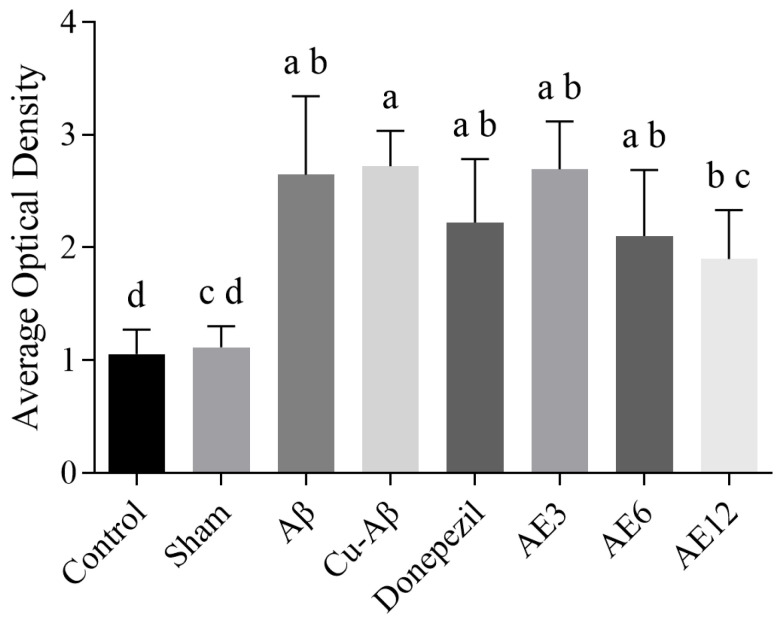
Effect of AE on the Aβ plaque in rat hippocampus. Mean values with the same superscript letters are not significantly different, whereas those with the different superscript letters are significantly (*p* < 0.05) different (*n* = 3).

As shown in Figure 5A,B, no Aβ aggregates were observed in the hippocampus of the control and sham group, whereas there were conspicuous brownish-yellow plaques in hippocampal areas of model groups (Figure 5C,D, black arrows) and abundant brownish-yellow granules around the neurons at the same time, which both represent deposition of Aβ. It showed less Aβ deposits in donepezil and all the doses of the AE group (Figure 5E–H, black arrows) compared to the hippo-CuAβ group. Moreover, we conducted quantitative analysis using ImageJ to make the results clearer. There was a significant difference in the high-dose AE group compared to the hippo-CuAβ group, while the donepezil group had no significant differences (Figure 6). Though the other dose groups did not show statistical differences, there was an obvious decreasing trend in the three treatment groups, confirming that AE effectively reduced Aβ deposition in the hippocampus of AD-like model rats, especially high-dose AE.

### 3.4. AE Decreased Copper Levels in Brain of AD-like Model Rats

#### 3.4.1. Method Validation

The standard curves for determination of copper content in serum and brain both indicated a strong linear relationship from 0 to 400 μg/L. The equations for the two relationships were as follows: y = 28.805x + 0.0423 (R2 = 0.998) and y = 0.0021x + 0.0016 (R2 = 1.000), respectively. For serum samples, the detection limit (LOD) was 1.357 ng/mL. When standard additions were made, the recovery rate ranged from 103.9% to 108.1% and relative standard deviation (RSD) ranged from 0.39% to 1.98%. Similarly, for brain tissue samples, LOD was 0.143 ng/mL, recovery rate ranged from 85.9% to 112.8%, and RSD ranged from 0.86% to 0.94%. The recovery of reference material met the acceptance criteria defined as 85% or more for higher levels of quality control standards, and precision was also acceptable with variability well below the acceptance criteria of 5%. Based on these data, the method we used was suitable as a reference method for assigning values of copper in serum as well as brain samples.

#### 3.4.2. Quantitative ICP-MS Measurements

**Figure 7:**
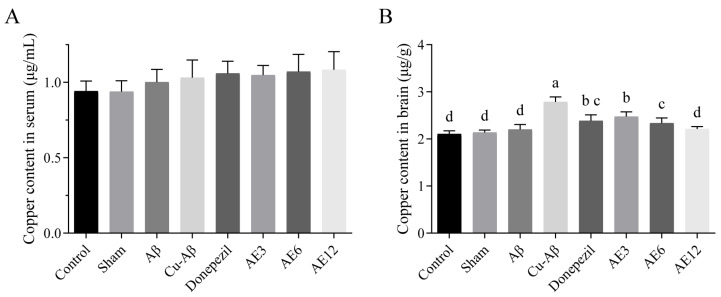
Effect of AE on the copper content in serum and brain tissue. (**A**) Copper content in serum (*n* = 8). (**B**) Copper content in the brain. Mean values with the same superscript letters are not significantly different, whereas those with different superscript letters are significantly (*p* < 0.05) different (*n* = 5).

As shown in Figure 7A, they were non-significantly different to copper content in serum samples among all groups, while a statistically significant increase in the brain samples of the hippo-CuAβ group was detected compared to the sham group, which was as expected (*p* < 0.05, Figure 7B). Also, significant effect of treatment, either donepezil or different doses of AE, was found on the reduction in copper levels of rat brain tissue compared to the hippo-CuAβ group (donepezil group, 14.03%; low-dose AE group, 11.15%; medium-dose AE group, 16.19%; high-dose AE group, 20.50%, all *p* < 0.05, Figure 7B). Particularly, the significant difference between donepezil and high-dose AE (*p* < 0.05, Figure 7B) emphasizes the effectiveness of AE. The difference in serum samples was non-significant. The above comprehensive results provided valuable insights into the excluded effect of AE to copper in brain of rats. In particular, the effect of AE on copper exclusion from the brain was dose-dependent.

### 3.5. AE Influenced the Expression of Protein Associated with Aβ Production and Transporter

#### 3.5.1. Protein Associated with Aβ Production

**Figure 8:**
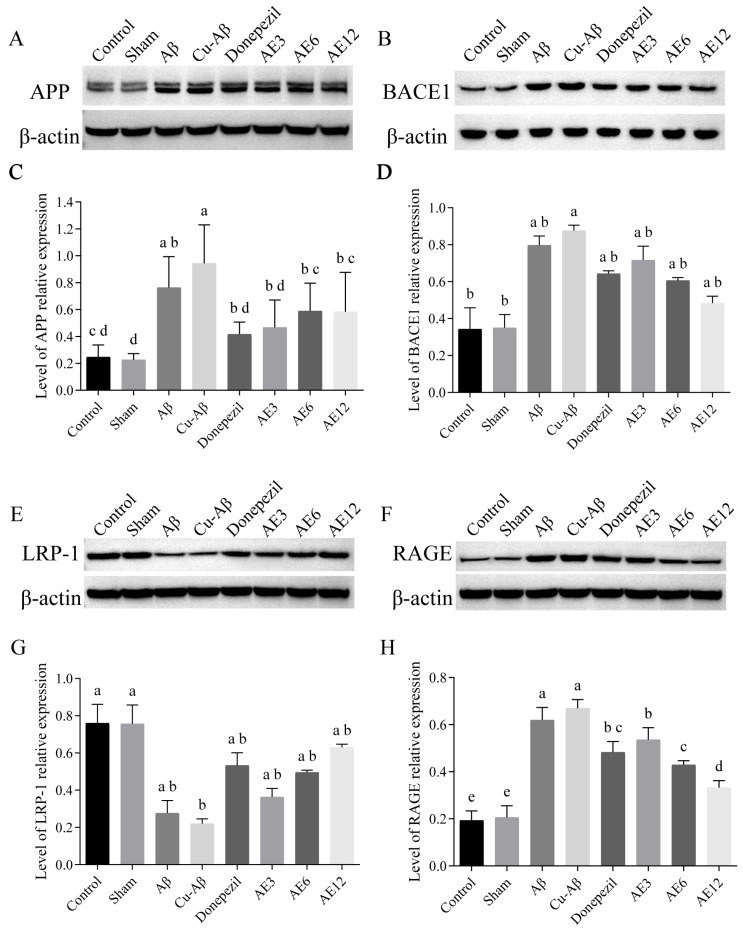
Effect of AE on the expression of protein associated with Aβ production and transporter. (**A**,**C**) Relative expression of APP in the rat brain tissue. (**B**,**D**) Relative expression of BACE1 in the rat brain tissue. (**E**,**G**) Relative expression of LRP-1 in the rat brain tissue. (**F**,**H**) Relative expression of RAGE in the rat brain tissue. Mean values with the same superscript letters are not significantly different, whereas those with the different superscript letters are significantly (*p* < 0.05) different (*n* = 3).

Compared with the sham group, there were higher levels of APP relative content in the hippo-Aβ group and hippo-CuAβ group (both *p* < 0.05, Figure 8C), while only the hippo-CuAβ group had statistical increases in BACE1 relative content (*p* < 0.05, Figure 8D) in brain homogenates, consistent with previous studies where the two protein had higher content in AD [37]. The relative expression of APP was significantly decreased in all the treatment groups, compared with the hippo-CuAβ group (all *p* < 0.05, Figure 8C). Also, for BACE1, a decreasing trend was observed in the treatment groups, though the decrease was not statistically significant (Figure 8D). The difference was probably due to the fact that the cleavage of APP at β-secretase sites was enhanced by BACE1 so that the content of APP was influenced on it, not only on the effect of AE [38].

#### 3.5.2. Protein Associated with Aβ Transporter

Compared with the sham group, we found significant increases in the hippo-Aβ group and hippo-CuAβ group of the relative expression of RAGE (both *p* < 0.05, Figure 8H), while only the hippo-CuAβ group showed a decrease in the relative expression of LRP-1 (*p* < 0.05, Figure 8G), as expected. Combined with the results in 3.5.1, it is demonstrated that the AD-like model rats did appear to have abnormal Aβ metabolism, where the participant of copper made matters worse. As shown in Figure 8E,F, there were obvious increased and decreased band strengths of LRP-1 and RAGE in visual Western blot results, confirming the quantitative densitometry analysis, where only the relative expression of RAGE decreased significantly in all treatment groups with the dose-dependent effect of AE, compared with the hippo-CuAβ group (all *p* < 0.05, Figure 8H). Both medium- and high-dose AE performed significantly better than donepezil in the decrease in the RAGE level (both *p* < 0.05). Our results indicated that the effect of AE on transport of Aβ was mainly reflected in the inhibitory effect of Aβ transport into brain.

### 3.6. AE Improved the Antioxidant Capacity of AD-like Model Rats

**Figure 9:**
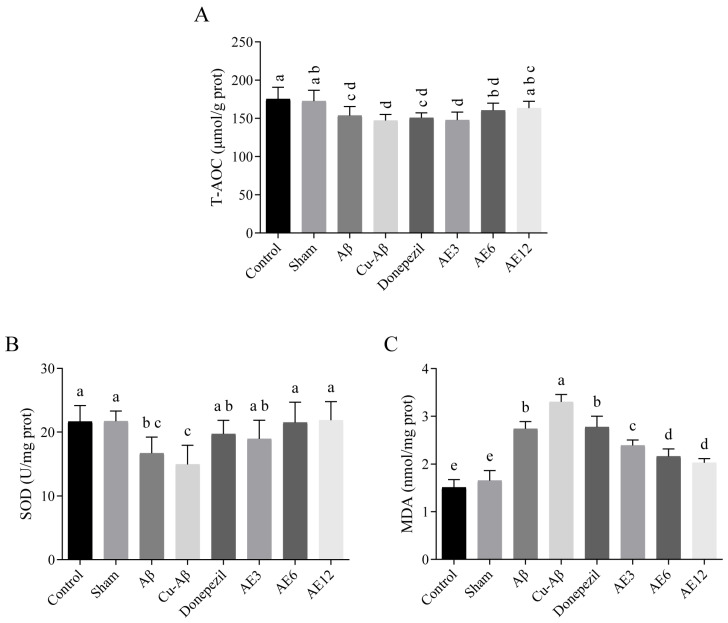
Effect of AE on the antioxidant capacity in hippo-CuAβ rats. (**A**) Level of T-AOC in the rat brain tissue. (**B**) Activity of SOD in the rat brain tissue. (**C**) Content of MDA in the rat brain tissue. Mean values with the same superscript letters are not significantly different, whereas those with the different superscript letters are significantly (*p* < 0.05) different (*n* = 5).

In our study, compared with sham group, there were significant alternations in the level of T-AOC, SOD activity, and content of MDA in model groups (all *p* < 0.05, Figure 9). And the MDA content increased much higher in the hippo-CuAβ model than in the hippo-Aβ model (*p* < 0.05). It follows from the above that the copper-Aβ complex caused damage to rat such that its antioxidant system was insufficient to resist OS and the increased degree of lipid peroxidation may be related to the inhibition of SOD activity and the untimely scavenging of free radicals. As expected, AE treatments reversed the effects of injections to varying degrees. In four treatment groups, there were increasing tendencies in T-AOC level and SOD activity compared with the hippo-CuAβ group. The tendency of three dose of AE in SOD activity showed statistical difference (all *p* < 0.05, Figure 9A,B), which indicated an obvious induction effect of AE on SOD activity in model rats to resist oxidative damage induced by copper-Aβ complex. And only high-dose AE could significantly increase the T-AOC level (*p* < 0.05), while donepezil could not. Also, for MDA content, there were fully significant decreases in all the AE treatment groups compared with two model groups (all *p* < 0.05, Figure 9C), and they were all statistically different with the donepezil group (all *p* < 0.05, Figure 9C). The reversed results of T-AOC and SOD and the reduction in lipid peroxidation product above verified that AE could restore the antioxidant capacity of CuAβ-induced AD-like model rats, thereby contributing to weakening the degree of OS.

### 3.7. AE Alleviated the Neuroinflammation in the Brain of AD-like Model Rats

**Figure 10:**
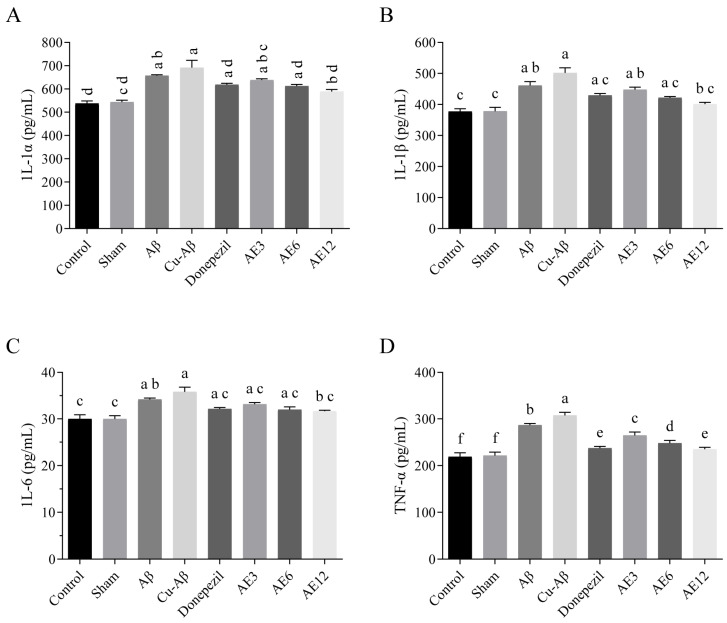
Effect of AE on the neuroinflammation in hippo-CuAβ rats. (**A**) Content of IL-1α in the rat brain tissue. (**B**) Content of IL-1β in the rat brain tissue. (**C**) Content of IL-6 in the rat brain tissue. (**D**) Content of TNF-α in the rat brain tissue. Mean values with the same superscript letters are not significantly different, whereas those with the different superscript letters are significantly (*p* < 0.05) different (*n* = 5).

In our study, compared to the sham group, the content in the brain of four kinds of pro-inflammatory cytokines all increased in model groups to varying degrees, especially for the hippo-CuAβ group (all *p* < 0.05, Figure 10), indicating that the injection of copper-Aβ complex induced neuroinflammation in the brain of rats. Notably, high-dose AE could significantly reverse the four indices (all *p* < 0.05, Figure 10), while donepezil could only significantly decrease the TNF-α (*p* < 0.05, Figure 10D). At the same time, there was a decreasing tendency in each index with the increase in the AE dose. These results are similar to those of previous reports showing that AE markedly suppressed the production of pro-inflammatory cytokines and exerted an anti-inflammatory effect through multiple mechanisms [20].

### 3.8. AE Regulated the Neurotransmitter Dyshomeostasis in the Brain of AD-like Model Rats

**Figure 11:**
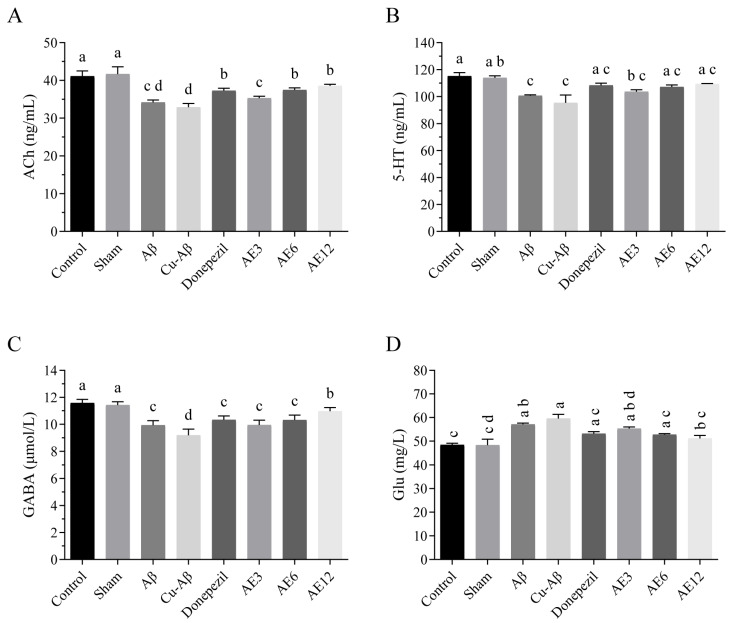
Effect of AE on the neurotransmitters in hippo-CuAβ rats. (**A**) Content of ACh in the rat brain tissue. (**B**) Content of 5-HT in the rat brain tissue. (**C**) Content of GABA in the rat brain tissue. (**D**) Content of Glu in the rat brain tissue. Mean values with the same superscript letters are not significantly different, whereas those with the different superscript letters are significantly (*p* < 0.05) different (*n* = 5).

In our study, a significant decrease in ACh, 5-HT, and GABA content and a significant increase in Glu content were detected in hippo-CuAβ model rats compared to the sham group (all *p* < 0.05, Figure 11). And three of them showed a statistical alternation after being treated with a high dose of AE (ACh, *p* < 0.05; GABA, *p* < 0.05; Glu, *p* < 0.05, Figure 11A,C,D). Expressly, high-dose AE performed significantly better than donepezil in the increase in GABA content (*p* < 0.05, Figure 11C). The previous study showed that rhubarb anthraquinone glycosides can effectively reduce the content of Glu and increase the content of GABA in the brain and colon tissue of rats, which is consistent with our findings [39]. It is well-known that the balance between GABA and Glu can avoid excitatory overstimulation and concurrent excitotoxic damage [40]. The glutamate decarboxylase (GAD) enzyme converts Glu to GABA while consuming a proton [41]. Therefore, the possible mechanism is that AE rescued Glu uptake or GAD level and activity, improved abnormal GABA and Glu content, and thus regulated the balance between excitatory amino acids and inhibitory amino acids. It has been demonstrated that AE exhibited an excellent acetylcholinesterase (AChE)-inhibitory activity in a dose-dependent manner in vitro [42]. This may be the reason why ACh content was reduced after AE treatment. Though further research is needed, current evidence showed that AE could reverse the abnormal neurotransmitters homeostasis in CuAβ-induced AD-like model rats.

## 4. Discussion

Copper is a serious contributor to cognitive impairment [8], and both histological and biochemical abnormalities in AD have been found to be associated with it [43]. Our results shows that AE administration can attenuate behavioral, histopathological, and biochemical implications in CuAβ-induced AD-like model rats, suggesting that AE is a neuroprotective agent against AD based on the principle of metal coordination.

First, through injection of Aβ monomer or copper-Aβ complex in the bilateral hippocampus of healthy male SD rats, we successfully constructed two kinds of nontransgenic AD-like animal models (a hippo-Aβ model and a hippo-CuAβ model). Of the two, the hippo-CuAβ model showed more serious cognitive impairments (though not statistically significantly so), more extensive neuronal apoptosis and Aβ deposition in the hippocampal region, and more severe deregulation of copper in the brain (*p* < 0.05). This suggests that the hippo-CuAβ model can be used as a robust nontransgenic model to mimic and recapitulate AD-like symptoms, which is consistent with the predecessors’ study [44]. Moreover, numerous animal models of neurotoxicity have been developed by copper-intoxication [45,46], emphasizing the relationship between high copper levels and the AD pathology. However, this modeling method cannot simulate the process of copper ions crossing the blood–brain barrier and accumulating throughout various regions of the brain in AD patients under natural conditions. For example, copper imbalance could be achieved by specifically knocking down or overexpressing the genes related to metal ion homeostasis [47].

Next, we investigated the effect of AE on CuAβ-induced AD-like model rats and the mechanism of action through different methods, including behavioral, histopathology, and biochemical experiments. In the results, the administration of AE significantly improved the behavioral performance of the hippo-CuAβ rats: higher spontaneous alternation ratio in the YMT, more exploration behaviors in the OFT, and a greater ability to learn and consolidate new memories in the MWM.

Furthermore, our histopathological results showed that AE attenuated neuronal apoptosis and reduced Aβ deposition in the hippo-CuAβ group. We believe that statistically insignificant differences in Aβ deposits between the two model groups are due to the short time interval between modeling and testing. In fact, the deposits in the CuAβ hippocampuses were distributed more widely than in the Aβ group, which is consistent with previous research [35]. Although we were not able to demonstrate the induction effect of copper on Aβ deposition, previous research has already confirmed that copper has a high affinity to bind with Aβ, which results in Aβ aggregation [12,48]. Our future studies may focus on the molecular evidence for copper-induced Aβ aggregation and the characterization of the Cu-Aβ complex [49]. For example, comprehensive three-dimensional morphometric analysis of Aβ plaques could be monitored by X-ray phase-contrast tomography [50].

We also found that AE treatment significantly decreased the copper content of the brain tissues. However, the effect of AE on copper exclusion from the brain requires further verification by histological evidence. For instance, the immunoreactive staining techniques for metallothionein could be used to indirectly reflect the location of copper deposits [51]. Also, copper-related evidence focused on the in vivo biotransformation of AE is necessary. Moreover, the statistically insignificant difference in copper content in serum samples may be due to copper excretion out of the body to some extent so that the actual copper content cannot be measured accurately in serum. This inspired an idea for future research to clarify the copper exclusion mechanism of AE. For instance, the form of copper in the excreta could be monitored by HPLC-ICP-MS, and the blood, urine, and feces of copper-induced AD rats could be assayed by LC-MS/MS to determine whether there is AE–copper complex in vivo.

In addition, we explored the expression of proteins related to the synthesis and transport of Aβ. The observed increases in APP, BACE1, and RAGE, and the decrease in LRP-1 may be responsible for the enhanced flux of abnormal produced Aβ across the BBB into the brain of the model rats. AE treatment could play an inhibitory role in the whole process by reducing Aβ deposition in the brain through indirect and direct pathways. Indirectly, it could affect the expression of proteins related to Aβ metabolism, thereby reducing the production and inhibiting the transport into the brain. Directly, it could interact with copper in the brain and interfere with the stability of copper-Aβ species to reduce the amount of toxic Aβ deposition.

Finally, we investigated types of biochemical indices related to the Aβ cascade, including OS, neuroinflammation, and neurotransmitter homeostasis, and found that the copper-Aβ complex indeed worsened AD pathology, whereas AE can reverse these deteriorations. Combined with the reported pharmacological effects of AE [52], we believe that not only the direct effects of AE but also the reduction in copper-Aβ complex contribute to these results [53,54]. This is because the copper-Aβ complex can lead to severe ROS accumulation through different mechanisms, including the cyclic generation of hydrogen peroxide and the mitochondria dysfunction [14,17].

The difficulty in directly extracting metal ions from metalated Aβ is considered to lie at the origin of the rupture of the metal homeostasis regulation in AD brains [9]. Some studies have therefore focused on the design of novel drugs as metal-chelating agents for the treatment of AD [55], which have been shown to inhibit metal-dependent Aβ aggregation [56,57,58]. Due to the chemical structural advantage discussed above, we hypothesized that AE could remove the metal ions from neurotoxic metal–Aβ complexes as a metal-chelating agent. There has already been some research to support our hypothesis. For example, the interaction of metal ions with anthraquinones has been used for drug transport. This is because the metal–anthraquinone complex structure disintegrates due to the sensitivity at low pH, which in turn releases the ligand drug molecules [59]. Moreover, formations of AE and metal ion complexes have been found to exist, including the AE–copper (II), the AE–iron (II), and the AE–magnesium (II) complexes, which have been shown to enhance antioxidant activity to a greater extent than the AE itself [60,61]. Additionally, we have ourselves already shown that AE can inhibit copper-induced Aβ aggregation and alleviate neurotoxicity induced by the copper-Aβ complex on SH-SY5Y human neuroblastoma cells in a previous in vitro study [62] and have also demonstrated the ameliorating effect of AE against aluminum-induced AD model rats in another previous study [63].

According to the results of experiment, it can be concluded that AE interacted with copper ions to inhibit the toxicity of the copper-Aβ complex, thereby achieving multiple effects to ultimately ameliorate the pathological manifestations of AD. The strategy of using active ingredients of natural medicines as metal coordination ligands is anticipated to inspire the development of potential therapeutic candidates to treat AD in the near future. However, its long-term toxicity and systemic safety need to be further studied.

## 5. Conclusions

AE efficiently reversed memory impairment and damage to hippocampus nerve cells in CuAβ-induced AD-like model rats constructed by bilateral hippocampal injection, which was attributed to its effects on copper and the Aβ pathological cascade. Considering other AD-targeting drugs with single pharmacological effects, this paper provides promising results for the broad application prospects of AE as a kind of copper coordination agent against AD.

## Figures and Tables

**Table 1:** Anxiety of rats in each group evaluated by the natural preference of center zone in the Open Field Test.

Groups	Number of Entries in the Center Zone (Times)	Duration in the Center Zone (s)
control group	7.75 ± 2.60 ^a^	28.56 ± 9.71 ^a^
sham group	7.38 ± 2.07 ^ab^	23.78 ± 5.38 ^a^
hippo-Aβ group	2.25 ± 1.28 ^d^	5.69 ± 1.58 ^c^
hippo-CuAβ group	2.13 ± 1.13 ^d^	3.36 ± 1.88 ^c^
donepezil group	5.25 ± 1.98 ^c^	12.95 ± 4.38 ^ac^
low-dose AE group	3.00 ± 1.85 ^d^	6.87 ± 1.66 ^bc^
medium-dose AE group	5.63 ± 2.00 ^bc^	12.54 ± 2.38 ^ac^
high-dose AE group	7.75 ± 2.32 ^a^	17.81 ± 3.59 ^ab^

Mean values with the same superscript letters are not significantly different, whereas those with different superscript letters are significantly (*p* < 0.05) different (*n* = 8).

## Data Availability

The data presented in this study are available in Science Data Bank at https://doi.org/10.57760/sciencedb.24018 (accessed on 12 January 2026). Further inquiries can be directed to the corresponding author.

## Supplementary Materials

The following supporting information can be downloaded at https://www.mdpi.com/article/10.3390/cimb48010086/s1.
