# Supplementary material for: Behavioral, Histopathological, and Biochemical Implications of Aloe Emodin in Copper-Aβ-Induced Alzheimer’s Disease-like Model Rats
Source: Curr Issues Mol Biol. 2026 Jan 15;48(1):86. doi: 10.3390/cimb48010086 (PMC12840291; doi:10.3390/cimb48010086)
Supplement: Supplementary file 1 [file cimb-48-00086-s001.zip › cimb-4031327-supplementary.pdf]

Supplementary Materials

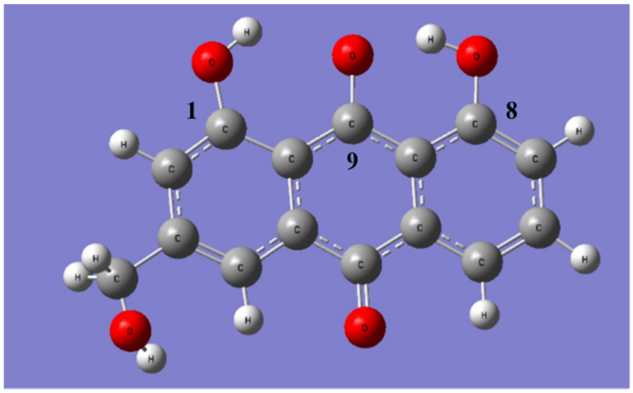

Figure S1. Chemical structure of aloe emodin. The image was created using GaussView 5.0.9 software.

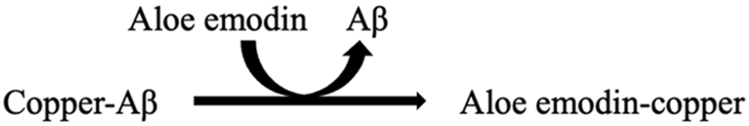

Figure S2. The possible mechanism of aloe emodin in AD based on the metal coordination principle.

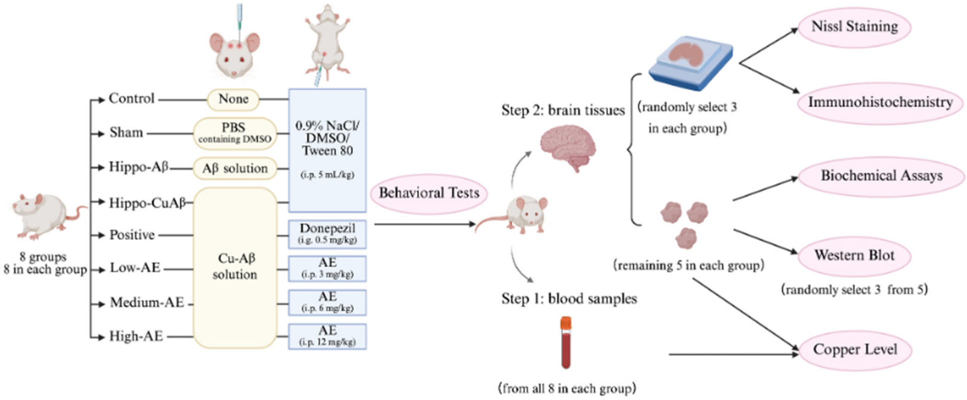

Figure S3. Schematic diagram of the experimental process.

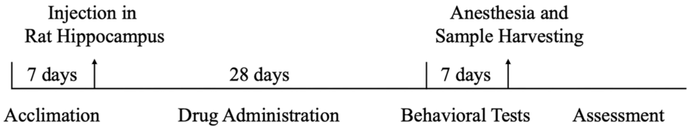

Figure S4. Schematic diagram of the experimental timeline.
